# Surface roughness and dimension accuracy data from hybrid manufacturing process using PLA material

**DOI:** 10.1016/j.dib.2024.110477

**Published:** 2024-04-25

**Authors:** Benny Susanto, Muhammad Ibnu Rashyid, Fefria Tanbar, Hifni Mukhtar Ariyadi, Muhammad Akhsin Muflikhun

**Affiliations:** a PLN Research Institute, Jakarta, Indonesia; b Mechanical and Industrial Engineering Department, Gadjah Mada University, Indonesia

**Keywords:** Hybrid manufacturing, 3D printing, Surface roughness, Dimension accuracy

## Abstract

This paper introduces a comprehensive dataset focusing on the surface roughness and dimensional accuracy of 3D printed specimens derived from a hybrid manufacturing process. The design of these specimens incorporates surfaces oriented at 0˚, 45˚, and 90˚ angles for surface roughness testing, along with cylindrical, radial, and pocket areas to evaluate dimensional accuracy. Utilizing PLA material, the specimens undergo a printing phase followed by milling within the same machine, thereby enhancing both surface roughness and dimensional quality. Surface roughness data is gathered through a surface roughness tester, while dimensional accuracy is assessed using a digital vernier caliper. The dataset includes comparative analyses conducted before and after the hybrid manufacturing process, revealing notable improvements in both surface roughness and dimensional accuracy post-processing. These findings furnish valuable insights for researchers and engineers engaged in hybrid manufacturing processes involving PLA material, serving as a foundational resource for further investigations and advancements in the field.

Specification Table

**Table:** 

Subject	Engineering
Specific subject area	Hybrid manufacturing, 3D printing and milling process, Surface roughness, Dimension accuracy.
Type of data	1.Tables
	2. Figures
Data collection	Surface roughness raw data obtained from the surface roughness tester is measured in Ra (arithmetic average) with the unit of μm. The raw data output from the surface roughness tester is in surface length (mm) and surface roughness (μm). The data obtained also shows the surface roughness shape shown on the graph. Dimension accuracy data were obtained from a digital vernier calliper. Each dimension was measured 3 times.
Data source location	Data were obtained from the Advanced Manufacturing lab, department of Mechanical and Industrial Engineering, Gadjah Mada University
Data accessibility	With the Article
Related research article	

## 1 Value of the Data


 

• The data presented in this study provide comprehensive surface roughness values at 0˚, 45˚, and 90˚ angles for both printed-only specimens and PLA specimens that underwent both printing and milling processes.
• Dimension accuracy data of PLA are useful for the estimation of shrinkage on printed-only specimens as the basis to resize the specimen for the hybrid manufacturing process.
• Surface roughness and accuracy data are useful for several studies fields such as sustainable manufacturing using 3D printing, optimizing 3D printed products with milling post-processing, and improving the quality of 3D printed products.
• Differences in roughness values between 3D printing only and hybrid manufacturing are beneficial for assessing component performance in terms of friction, wear, and sealing capabilities.

## 2 Background

The manufacturing process of 3D printing presents inherent challenges such as higher surface roughness and low accuracy values, primarily attributable to the layer-by-layer construction method and material shrinkage up to around 0.5 mm [1,2]. This variability in surface characteristics and dimensional accuracy is influenced by factors such as layer thickness and printing angles, which necessitates careful consideration during the printing process [3,4]. However, these challenges can be solved by post-processing techniques such as subtractive machining offer viable solutions to enhance the quality of printed objects. Adjustments are needed to the specimens for post-processing wherein each dimension is expanded by 0.5 mm allowing ample room for subsequent milling procedures to precisely remove excess material. The critical importance of maintaining optimal surface roughness and dimensional accuracy cannot be overstated, especially in the production of high-precision products where even minor deviations can significantly impact performance and functionality. Thus, integrating effective post-processing methodologies into the 3D printing workflow is essential for achieving the desired level of product quality and reliability. This paper presented a comprehensive dataset of surface roughness in 0˚, 45˚, and 90˚ and dimensional accuracy from both printed-only and print milling specimens.

## 3 Data Description

In this paper, comprehensive raw data on surface roughness and dimensional accuracy are documented. Table 1 shows the specimen list. The specimens have 2 variations of layer thickness with the printed only designated by NM and printing-milling by M. The following numbers on the designation were the layer thickness with 0.1 mm and 0.2 mm. On the surface roughness raw data contains measurement length and surface roughness value. Visual comparisons of surface morphology, depicted in Fig. 2, Fig. 3, Fig. 4, showcase the distinct surface profiles at varying angles of 0˚, 45˚, and 90˚ showing the effect of manufacturing orientation. The comparison between pre- and post-hybrid manufacturing processes unveils significant differences in surface morphology, underscoring the profound impact on surface roughness values. Moreover, dimensional accuracy evaluations encompass measurements along the X, Y, and Z axes in relation to manufacturing orientation on 3D printer machines. This multifaceted approach to data analysis provides a comprehensive understanding of surface roughness and dimensional accuracy assessment of all samples as shown in Table 2, laying a solid foundation for further exploration and refinement in hybrid manufacturing methodologies.

**Table 1:** List of specimens.

Specimen designation	Layer Thickness (mm)	Printing speed (mm/s)	Endmill diameter (mm)	RPM (rpm)	Piece (pcs)
NM 0.2	0.2	60	–	–	3
M 0.2	0.2	60	6	5000	3
NM 0.1	0.1	60	–	–	3
M 0.1	0.1	60	6	5000	3
Total specimens					12

**Table 2:** Dimensional measurement.

Specimen		d1	d2	d3	d4	d5	d6	d7	d8
		mm							
NM 0.1	I	99.80	29.85	10.13	24.27	24.58	9.98	4.92	45.10
		99.83	29.97	10.08	24.32	24.58	9.98	5.08	44.95
		99.85	29.89	10.10	24.37	24.59	9.99	5.02	45.15
	II	99.96	29.97	10.00	24.77	24.66	10.07	4.95	45.00
		99.87	30.01	10.00	24.74	24.68	9.95	4.90	45.05
		99.97	30.04	9.94	24.78	24.70	9.90	5.02	44.95
	III	99.90	29.99	9.86	24.74	24.67	10.10	5.05	44.95
		99.90	30.00	9.99	24.76	24.64	9.91	5.06	45.20
		99.87	29.97	9.94	24.74	24.70	9.95	5.02	45.05

NM 0.2	I	99.90	29.93	9.85	24.65	24.73	9.86	5.00	45.40
		99.93	30.00	9.83	24.64	24.75	9.95	5.09	45.35
		99.80	29.94	9.93	24.63	24.77	10.01	4.99	45.05
	II	99.91	29.96	10.04	24.53	24.60	9.95	5.01	45.40
		99.92	30.07	10.02	24.54	24.63	9.75	5.09	45.35
		99.92	29.98	10.03	24.50	24.63	9.99	4.96	45.05
	III	99.78	29.86	10.02	24.61	24.72	9.93	5.02	45.40
		99.73	29.95	10.04	24.66	24.75	10.09	4.95	45.30
		99.81	29.90	9.93	24.68	24.75	10.22	4.96	45.50

M 0.1	I	100.04	30.00	10.00	24.97	24.95	9.95	4.97	44.95
		100.02	29.99	9.97	24.96	24.92	9.97	5.03	45.00
		100.03	29.98	10.07	24.98	24.93	10.05	5.07	45.20
	II	99.98	30.04	10.00	24.98	24.92	9.96	5.00	44.95
		100.00	30.04	10.05	24.99	24.94	10.00	5.01	45.00
		100.02	30.01	9.96	24.95	24.95	10.03	4.97	45.00
	III	99.97	30.00	10.00	24.94	24.95	9.96	5.09	45.05
		100.00	29.98	9.98	24.95	24.94	9.97	4.96	44.95
		100.00	30.01	10.01	24.93	24.98	9.99	4.99	45.00

M 0.2	I	100.04	30.04	9.99	25.04	24.96	9.98	4.99	45.05
		100.00	30.05	9.94	25.03	24.97	10.02	4.98	45.15
		100.01	29.98	9.95	25.04	24.96	10.02	5.01	44.90
	II	99.99	30.07	9.99	25.02	25.02	9.95	4.97	45.10
		100.01	30.03	10.04	25.01	24.99	9.96	4.98	45.05
		99.98	30.03	9.95	25.05	24.98	10.02	5.02	45.00
	III	100.02	30.04	9.99	25.04	25.02	9.97	4.98	45.00
		99.97	30.00	10.05	25.06	25.00	10.01	5.01	45.05
		99.93	29.99	9.94	25.03	25.07	9.96	4.97	44.95

## 4 Experimental Design, Materials, and Method

The material extrusion 3D printer operates on a layer-by-layer manufacturing principle, inherently leading to elevated surface roughness on printed objects. The PLA material has a low melting point, and environmentally friendly properties make it a popular choice among the 3D printing community. However, despite its advantages, PLA is susceptible to shrinkage with a dimensional error of ±0.5 mm compared with other method such as laser powder bed fusion (LPBF) [5,6]. Fortunately, post-processing techniques offer avenues to mitigate surface roughness issues. Among these techniques, milling has emerged as a viable option, with previous studies yielding promising results [7,8]. Moreover, employing milling post-processing not only addresses surface roughness concerns but also contributes to enhancing dimensional accuracy. Encouraged by the positive outcomes, researchers have explored novel approaches, such as the modification of CNC milling platforms, to integrate hybrid manufacturing methodologies [[9], [10], [11], [12], [13]]. This research, adapts conventional 3D printer platforms to incorporate milling capabilities, aiming to demonstrate comparable performance to dedicated CNC milling machines. A regular 3D printer platform underwent modification to facilitate milling operations on a secondary carriage, affirming its potential to achieve similar outcomes to CNC milling machine platforms. This innovative approach underscores the continuous evolution and adaptation within the realm of additive manufacturing, as researchers seek to optimize processes and broaden the scope of hybrid manufacturing techniques.

**Fig. 1:**
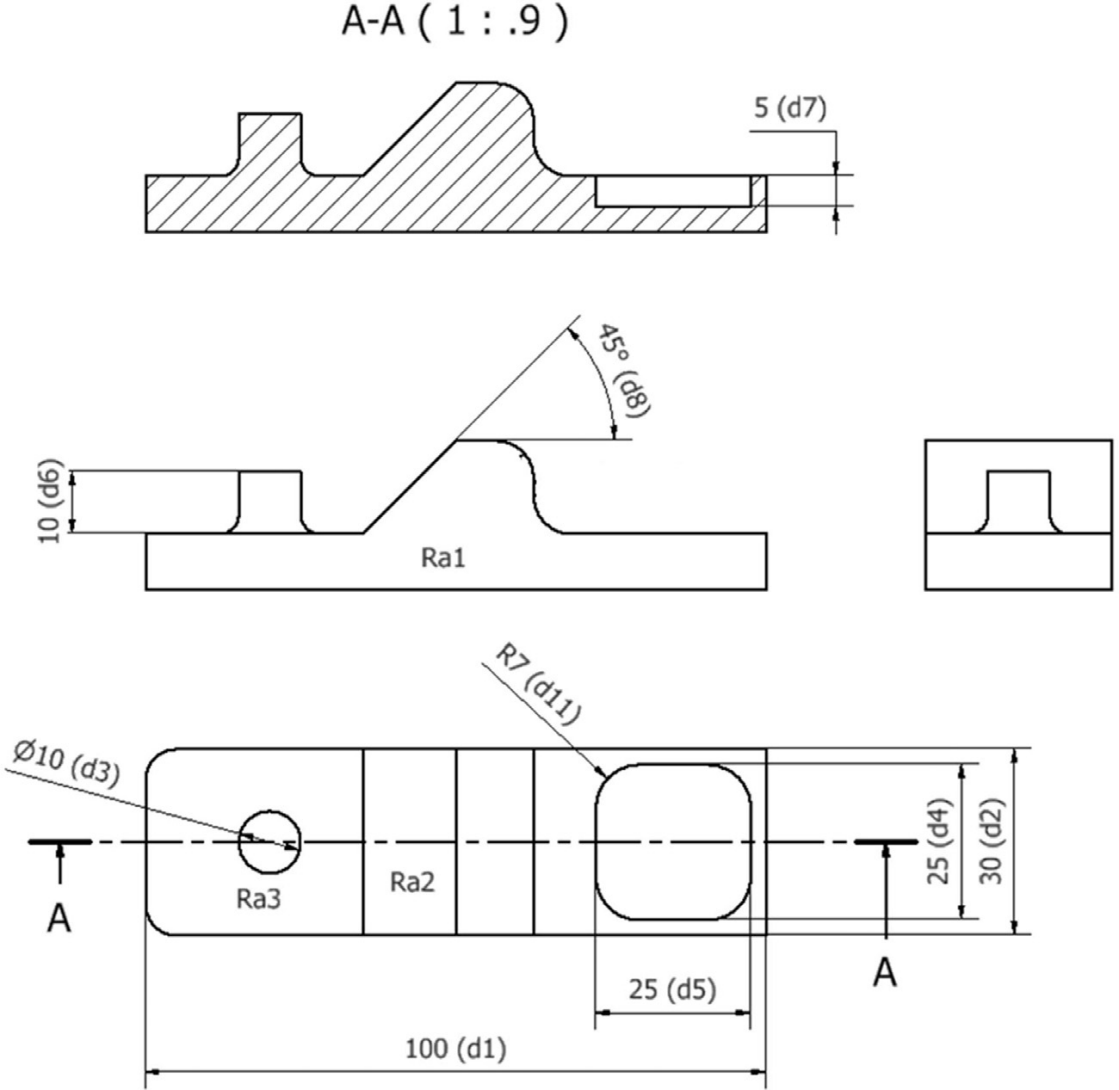
Specimen dimension and area of measurement.

The details regarding surface roughness area and specimen design are shown in Fig. 1. Surface roughness measurements, are designated as Ra1, Ra2, and Ra3. While dimensional measurements are represented by d1, d2, d3, d4, d5, d5, d7, and d8. The experimental and testing procedures are detailed in Fig. 5. The specimens underwent two manufacturing processes with two-layer thickness variation (0.1 mm and 0.2 mm), solely printing (NM 0.1 and NM 0.2) and printing followed by milling (M 0.1 and M 0.2). Each specimen was manufactured by 3 pieces, resulting in a total of 12 specimens. Surface roughness testing was conducted using ISO 1997 standards on surfaces oriented at 0˚, 45˚, and 90˚ with five surface tests performed for each area using the Mitutoyo SJ-210 Surface Roughness Tester. The evaluation parameter of surface roughness was Ra with a unit of μm with a measurement length of 3.2 mm and a measurement speed was 0.5 mm/s. On the 0˚ surface, surface tests were randomly positioned, whereas on the 45˚ and 90˚ surfaces, tests were perpendicular to the layer lines, more detailed information can be seen in Fig. 6. Dimensional accuracy assessments were carried out three times for each dimension using the Mitutoyo Digital Vernier Caliper, with dimensional accuracy categorized into X, Y, and Z axes corresponding to the printing orientation

**Fig. 2:**
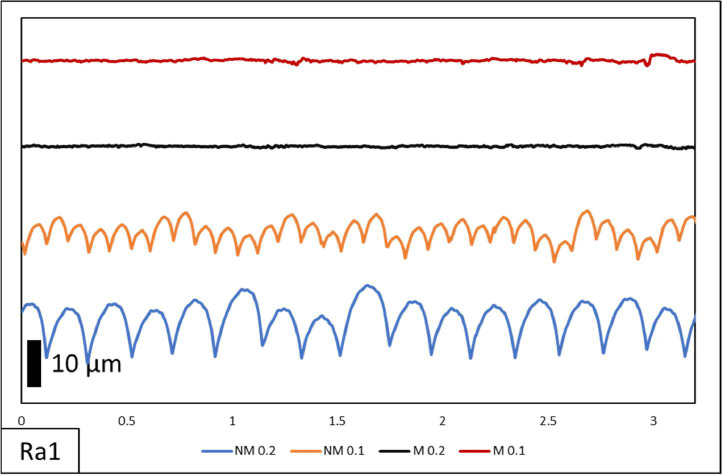
Ra1 of NM 0.1, NM 0.2, M 0.1 and M 0.2 surface roughness morphology.

**Fig. 3:**
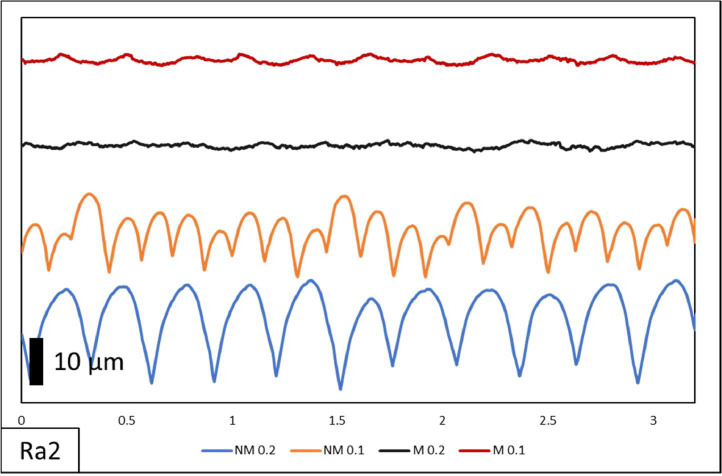
Ra2 of NM 0.1, NM 0.2, M 0.1 and M 0.2 surface roughness morphology.

**Fig. 4:**
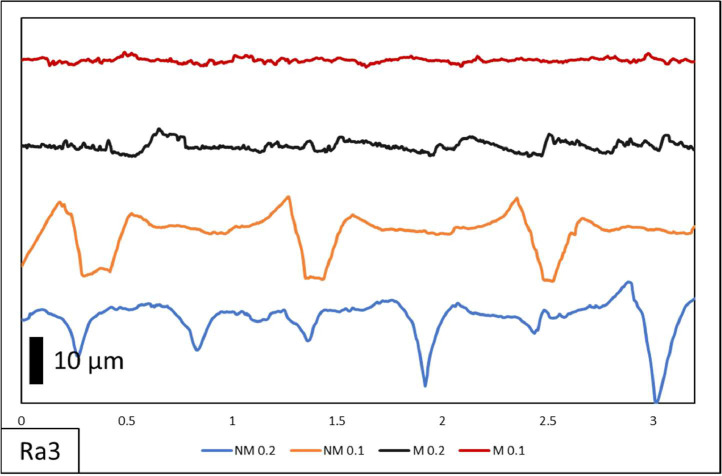
Ra1 of NM 0.1, NM 0.2, M 0.1 and M 0.2 surface roughness morphology.

**Fig. 5:**
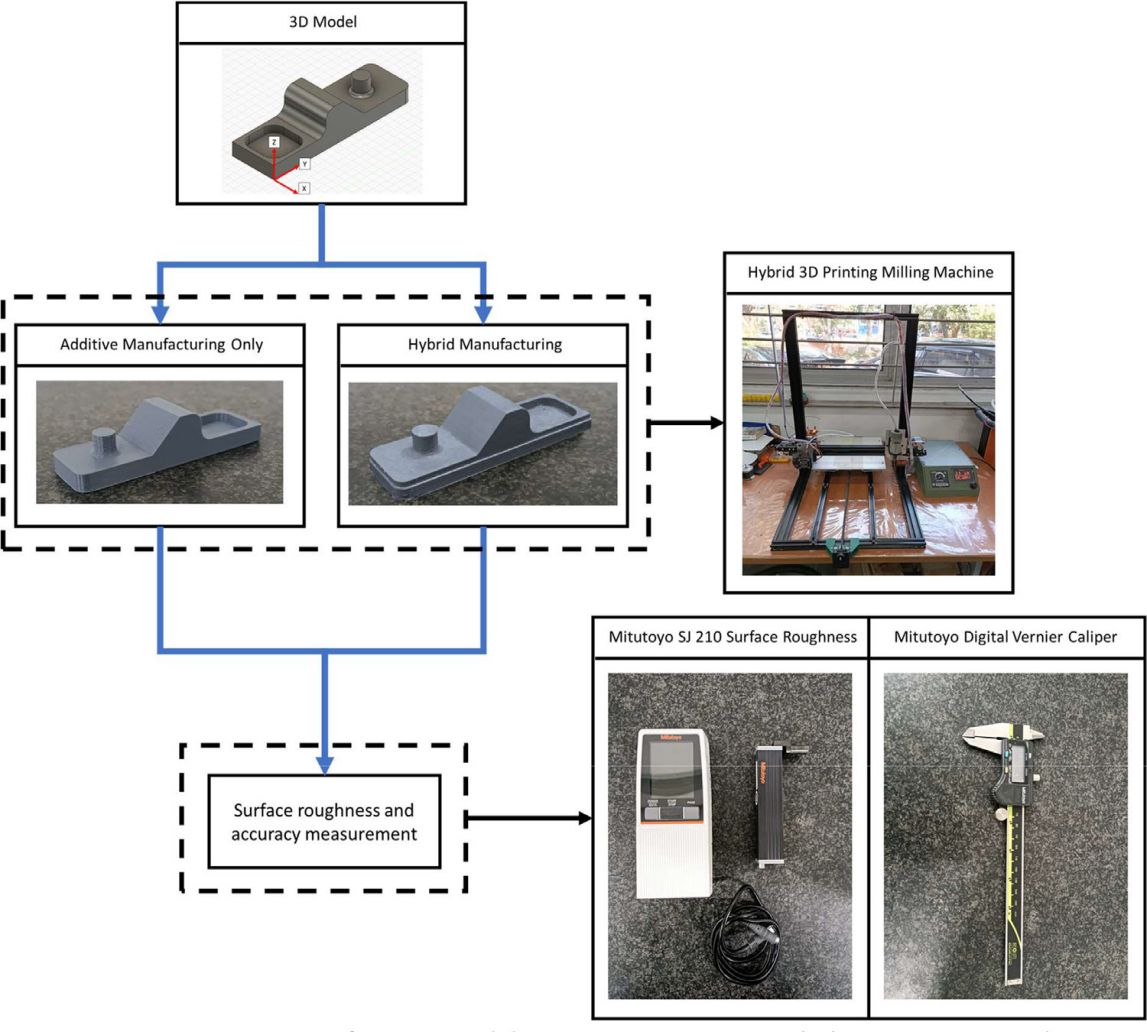
Specimen manufacturing and data acquiring process with the equipment used.

**Fig. 6:**
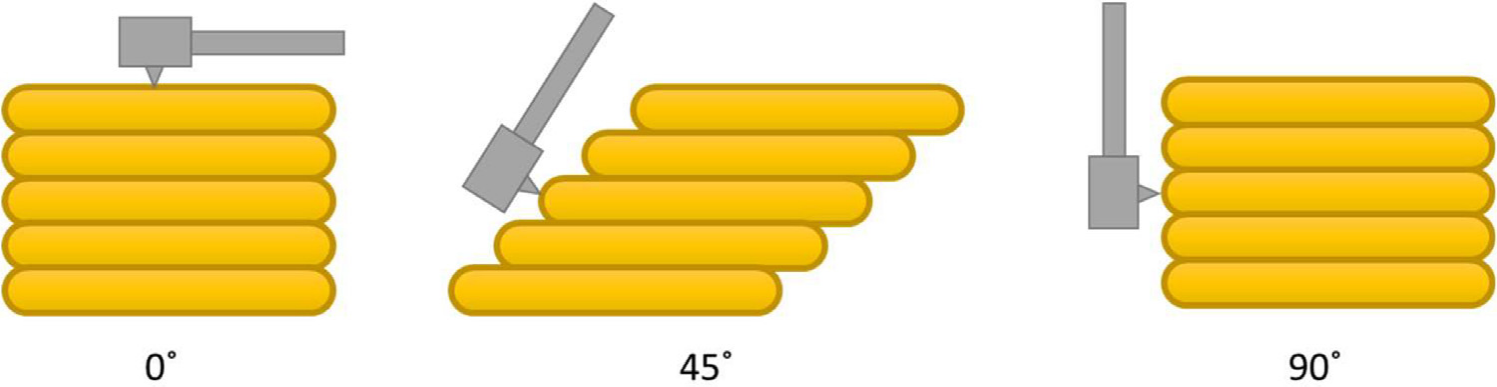
Surface roughness testing orientation.

## Limitations

Certain limitations were found during the data acquisition. The data from the 0˚ plane is influenced by the machining area. Certain parts may exhibit high or low values of surface roughness due to defects in the unfilled layers revealed after the milling processes. These unfilled layers can lead to higher roughness values, thereby increasing the total surface roughness. To avoid this, correct printing parameters must be established initially, ensuring that the layers are not under-extruded.

## Ethics Statement

The current work does not involve human subjects, animal experiments, or any data collected from social media platforms.

## CRediT authorship contribution statement

**Benny Susanto:** Conceptualization, Methodology, Investigation, Formal analysis, Software, Data curation, Validation, Visualization, Writing – original draft, Writing – review & editing. **Muhammad Ibnu Rashyid:** Conceptualization, Methodology, Investigation, Formal analysis, Software, Data curation, Validation, Visualization, Writing – original draft, Writing – review & editing. **Fefria Tanbar:** Writing – original draft, Writing – review & editing. **Hifni Mukhtar Ariyadi:** Writing – original draft, Writing – review & editing. **Muhammad Akhsin Muflikhun:** Conceptualization, Methodology, Writing – original draft, Writing – review & editing, Supervision, Funding acquisition, Resources, Project administration.

## Data Availability

Surface Roughness Data-1 (Original data) (Figshare).
